# Autoantibodies to synapsin I sequestrate synapsin I and alter synaptic function

**DOI:** 10.1038/s41419-019-2106-z

**Published:** 2019-11-14

**Authors:** Anna Rocchi, Silvio Sacchetti, Antonio De Fusco, Silvia Giovedi, Barbara Parisi, Fabrizia Cesca, Markus Höltje, Klemens Ruprecht, Gudrun Ahnert-Hilger, Fabio Benfenati

**Affiliations:** 1 Center for Synaptic Neuroscience and Technology, Istituto Italiano di Tecnologia, Largo Rosanna Benzi 10, 16132 Genova, Italy; 2 IRCSS, Ospedale Policlinico San Martino, Largo Rosanna Benzi 10, 16132 Genova, Italy; 3 Department of Experimental Medicine, University of Genova, Viale Benedetto XV, 3, 16132 Genova, Italy; 4 Department of Life Science, University of Trieste, via Giorgieri, 5, 34127 Trieste, Italy; 5 Institute of Integrative Neuroanatomy, Charité – Universitätsmedizin Berlin, Corporate member of Freie Universität Berlin, Humboldt-Universität zu Berlin, and Berlin Institute of Health, Berlin, Germany; 6 Department of Neurology, Charité – Universitätsmedizin Berlin, Corporate member of Freie Universität Berlin, Humboldt-Universität zu Berlin, and Berlin Institute of Health, Berlin, Germany

**Keywords:** Mechanisms of disease, Cellular neuroscience, Multiple sclerosis, Autoimmune diseases

## Abstract

Synapsin I is a phosphoprotein that coats the cytoplasmic side of synaptic vesicles and regulates their trafficking within nerve terminals. Autoantibodies against Syn I have been described in sera and cerebrospinal fluids of patients with numerous neurological diseases, including limbic encephalitis and clinically isolated syndrome; however, the effects and fate of autoantibodies in neurons are still unexplored. We found that in vitro exposure of primary hippocampal neurons to patient’s autoantibodies to SynI decreased the density of excitatory and inhibitory synapses and impaired both glutamatergic and GABAergic synaptic transmission. These effects were reproduced with a purified SynI antibody and completely absent in SynI knockout neurons. Autoantibodies to SynI are internalized by FcγII/III-mediated endocytosis, interact with endogenous SynI, and promote its sequestration and intracellular aggregation. Neurons exposed to human autoantibodies to SynI display a reduced density of SVs, mimicking the SynI loss-of-function phenotype. Our data indicate that autoantibodies to intracellular antigens such as SynI can reach and inactivate their targets and suggest that an antibody-mediated synaptic dysfunction may contribute to the evolution and progression of autoimmune-mediated neurological diseases positive for SynI autoantibodies.

## Introduction

A large battery of autoantibodies directed to neuronal proteins have been discovered in sera and CSF of patients suffering from a variety of neurological diseases^1^. These autoantibodies target two main classes of antigens, namely cell surface and intracellular antigens. Autoantibodies directed against the amino-3-hydroxy-5-methyl-4-isoxazolepropionic acid receptors (AMPARs), the N-methyl-D-aspartate receptor (NMDAR) and the γ-aminobutyric acid (GABA) type B receptor belong to the first group and are frequently detected in the serum and cerebrospinal fluid (CSF) of affected subjects^2^. A number of studies have clearly demonstrated the direct pathogenic role of these autoantibodies. Of note, antibody removal and immunotherapy are effective treatments and promote a clinical improvement in the affected patients. In contrast, the pathogenic role of antibodies directed against intracellular antigens, such as anti-neuronal nuclear antibody type 1 (ANNA-1), glutamic acid decarboxylase (GAD65) and amphiphysin, remains a topic of debate^2^. Although evidence exists that antibodies to GAD65 and amphiphysin have pathogenic effects, a clear mechanism for antibody internalization and interaction with intracellular targets is still lacking^3–7^.

A new member of the continuously growing list of target autoantigens of anti-neuronal antibodies is the SV-associated protein Synapsin I (SynI). Synapsin I is a phosphoprotein that coats the cytoplasmic side of SVs and plays multiple roles in the regulation of SV trafficking between the RP and the readily releasable pool (RRP) and in the facilitation of the post-docking steps of release^8^. Nonsense and missense mutations in the gene encoding SynI have been associated with epilepsy, autism spectrum disorder (ASD) and intellectual disability in humans^9–12^. SynI autoantibodies have been identified in serum and CSF from patients suffering of various neurological disorders, including limbic encephalitis, multiple sclerosis, epilepsy, anxiety, depressive and bipolar disorders, but not in healthy controls^13,14^. However, no studies correlating the effect of these antibodies to the brain pathology have been published to date.

In the present study, we sought to investigate whether autoimmune mechanisms involving SynI autoantibodies influence the properties of synaptic transmission and whether a direct interaction between autoantibodies and the intracellular synaptic target occurs within nerve terminals. Using patient CSF and purified antibodies, we found that anti-SynI antibodies induced marked effects on neuronal network connectivity and activity including a decreased density of synaptic connections and an impairment of excitatory and inhibitory transmission. We revealed that internalization of anti-SynI antibodies into neurons occurs through a clathrin-mediated endocytic pathway via Fcγ II/III receptors, followed by interaction with the cytosolic antigen and change in SV density and clustering within nerve terminals. Interestingly all these effects phenocopy the SynI knockout (KO) phenotype and are occluded in SynI KO neurons. Our findings provide new insights into a unique immune-neuronal interaction and indicate a potential pathogenic role of SynI autoantibodies in promoting “*autoimmune synaptopathies*”.

## Results

### SynI autoantibodies from patient CSF impair excitatory and inhibitory synaptic transmission in hippocampal neurons

To investigate potential pathogenic effects of SynI autoantibodies, we treated primary neurons with the CSF of a limbic encephalitis and clinical isolated syndrome patients (herein referred to as LE-CSF and CIS-CSF, respectively) that were characterized by high titers of IgG and IgA autoantibodies against SynI^13,14^. As control, we pooled CSF from two patients with non-inflammatory neurological disorders that were negative for anti-neuronal autoantibodies (herein referred to as control CSF). The demographic and clinical data are reported in Supplementary Table 1^13,15^. To rule out the possibility that human CSF per se may induce changes in synaptic transmission in primary neurons, we compared frequency and amplitude of mEPSCs and mIPSCs in untreated neurons, or in neurons treated daily for 3 days with control CSF or commercial human IgG/IgA antibodies. None of the experimental conditions induced any change in the electrophysiological parameters (data not shown). Accordingly, treatment with control CSF was used as control condition throughout the study.

**Fig. 1:**
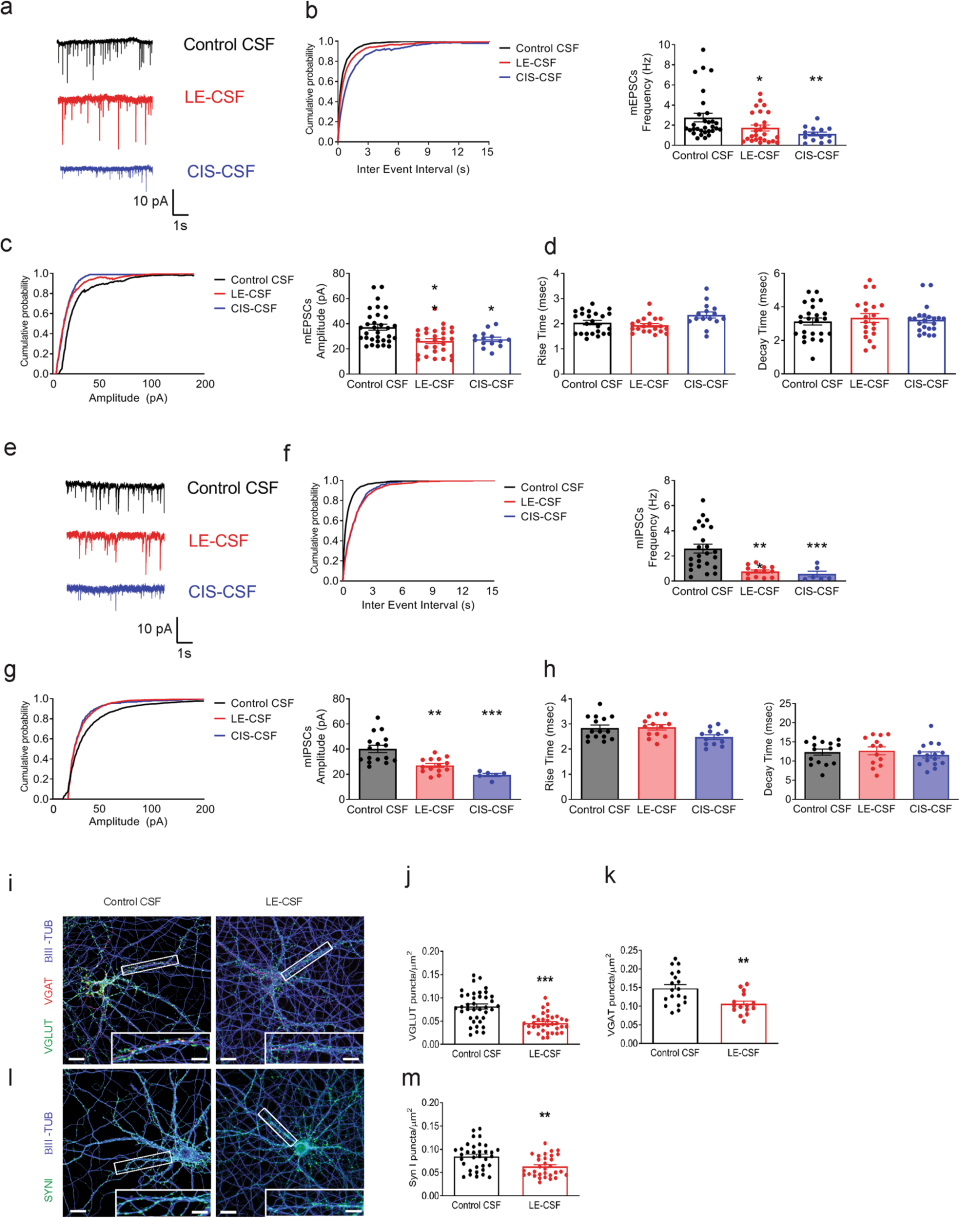
Patient-derived SynI autoantibodies decrease excitatory and inhibitory synaptic transmission and synapses. **a** Representative recordings of miniature excitatory postsynaptic currents (mEPSCs) in primary hippocampal neurons treated with either control CSF (1.5 μg/mL, black traces), LE-CSF (1.5 μg/mL, red traces) or CIS-SF (1.5 μg/mL, blue traces) for 72 h. **b** Cumulative distribution of inter-event intervals and mean mEPSC frequency. **c** Cumulative distribution and mean of mEPSC amplitude. **d** Rise and decay times of mEPSCs. **e** Representative recordings of miniature inhibitory postsynaptic currents (mIPSCs) in hippocampal neurons treated as in (**a**). **f** Cumulative distribution of inter-event intervals and mean frequency of mIPSCs. **g** Cumulative distribution and mean of mIPSC amplitude. **h** Rise and decay times of mIPSCs. Cumulative plots show pooled data from all neurons; graphs show means ± SEM, single neuron data points from at least three independent preparations; *p < 0.05, ***p < 0.001, Kruskal–Wallis test and Dunn’s post hoc. **i** Representative images of primary hippocampal neurons treated with either control CSF or LE-CSF (1.5 μg/mL) for 72 h and stained for β-III tubulin (blue), VGLUT1 (green) and VGAT (red). Scale bars, 10 and 1 μm for low and high magnification, respectively. **j**, **k** Density of VGLUT1-positive excitatory synapses (**j**) and VGAT-positive inhibitory synapses (**k**) in control CSF and LE-CSF treated neurons. **l** Representative images of primary hippocampal neurons treated as in (**a**) and stained for β-III tubulin (blue) and SynI (green). Scale bars, 10 and 1 μm for low and high magnification, respectively. **m** Quantification of the density of SynI immunoreactive synaptic puncta. Graphs show means ± SEM, single neuron data points from three independent preparations; **p < 0.01, ***p < 0.001, Mann–Whitney test.

We first addressed the effect of both LE-CSF and CIS-CSF treatments on excitatory synaptic transmission by recording mEPSCs in dissociated hippocampal neurons at 14 DIV after 72 h of incubation. Under this condition, the mEPSC frequency was significantly reduced in patient CSF-treated neurons as compared to control CSF and, consistently, the cumulative probability plots of inter-event intervals were shifted to greater values (Fig. 1a, b). mEPSC amplitudes and their the cumulative probability plot were also significantly decreased in neurons treated with LE-CSF and CIS-CSF (Fig. 1c), in the absence of changes in the rise and decay times (Fig. 1d). Next, we assessed inhibitory synaptic transmission by recording mIPSCs. Similar to excitatory transmission, we found a significant reduction in both mIPSC frequency and amplitude of in patient CSF-treated neurons compared to treatment with control CSF (Fig. 1e–g), in the absence of changes in the current kinetics (Fig. 1h). To exclude the possibility that the impairment of synaptic transmission was due to a nonspecific cytotoxic effect of the CSF, resting membrane potential, a very sensitive index of neuronal health, was measured by patch-clamp in the current-clamp configuration. No change in membrane potential was detected in neurons exposed to both LE-CSF and CIS-CSF as compared to control CSF-treated neurons, suggesting that neurons are healthy and viable (resting potential mV, mean ± SEM: control CSF −56,86 ± 1,57; LE-CSF −56.00 ± 1.05; CIS-CSF −51.33 ± 1635).

The consistent decrease in the frequency of spontaneous synaptic events of both excitatory and inhibitory transmission suggests that a possible loss of synaptic connection might have occurred during exposure to the patient-CSF. Indeed, double labeling with β-tubulin and either the glutamatergic or GABAergic SV markers VGLUT1 and VGAT demonstrated a significant decrease in the density of both excitatory and inhibitory synaptic puncta in cultures exposed to LE-CSF compared to control (Fig. 1i–j). Consistently, presynaptic labeling of both excitatory and inhibitory terminals with antibodies to SynI revealed a significant decrease of the SynI-positive puncta after LE-CSF treatment (Fig. 1i–j).

### The pathophysiological effects of patient’s CSF are specifically due to anti-SynI antibodies

**Fig. 2:**
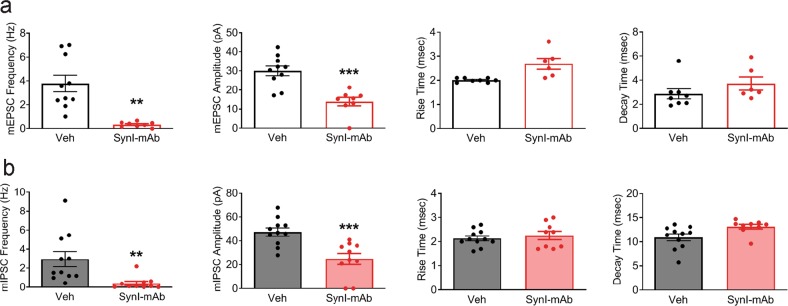
The synaptic effects of patient-derived SynI autoantibodies are mimicked by affinity-purified anti-SynI antibodies. **a**, **b** Mean frequency and amplitude of mEPSCs (**a**) and mIPSCs (**b**) in neurons exposed for 72 h to affinity-purified anti-SynI monoclonal antibodies (Synaptic System #106011; SynI-mAb, 1.5 μg/mL, red bar), as compared to vehicle (white bar). Graphs show means ± SEM, single neuron data points from three independent preparations; *p < 0.05, **p < 0.01, ***p < 0.001, Mann–Whitney test.

To unambiguously demonstrate that the physiological effects were causally linked to SynI autoantibodies, we used affinity-purified anti-SynI monoclonal IgGs (SynI-mAb). Interestingly, treatments with SynI-mAb induced a significant reduction in the frequency and amplitude of both mEPSCs and mIPSCs compared to control conditions (Fig. 2a, b), fully mimicking the results obtained with either LE-CSF or CIS-CSF (Fig. 1). These finding suggest that the impairment of excitatory and inhibitory synaptic transmission is specifically mediated by the autoantibodies to SynI.

### The expression of endogenous SynI is required for limbic encephalitis CSF-induced effects on synaptic transmission

**Fig. 3:**
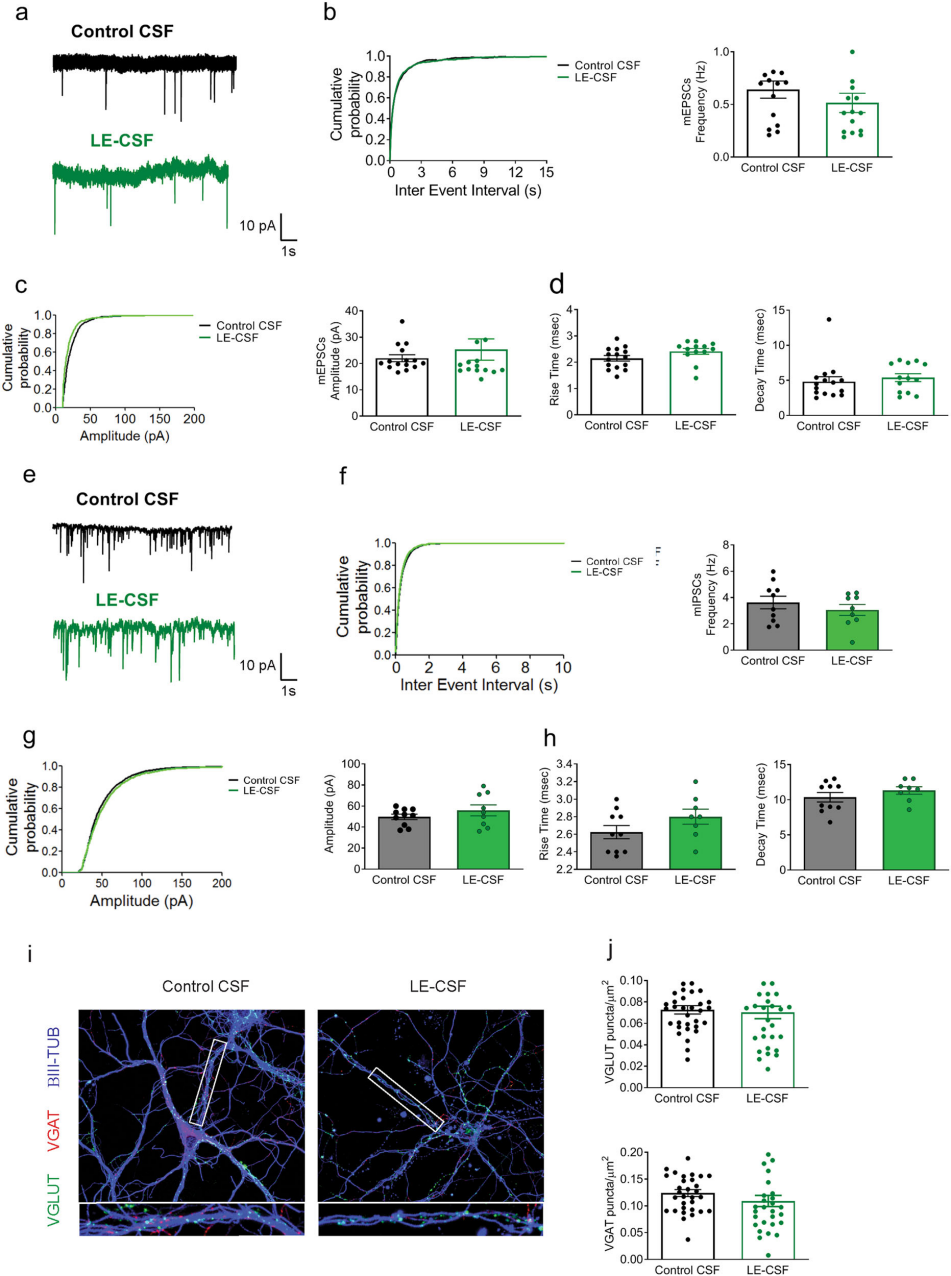
The effects of LE-derived SynI autoantibodies on excitatory and inhibitory transmission and synapses are abolished in SynI KO hippocampal neurons. **a** Representative recordings of mEPSCs in SynI KO hippocampal neurons treated with either control CSF (1.5 μg/mL, black trace) or LE-CSF (1.5 μg/mL, green trace) for 72 h. **b**–**d** Cumulative distribution of inter-event intervals and mean frequency of mEPSCs (**b**), cumulative distribution and mean of mEPSC amplitude (**c**) and rise/decay times of mEPSCs (**d**). **e** Representative recordings of mIPSCs in SynI KO hippocampal neurons treated as described in (**a**)**. f**–**h** Cumulative distribution of inter-event intervals and mean frequency of mIPSCs (**f**), cumulative distribution and mean of mIPSC amplitude (**g**) and rise/decay times of mIPSCs (**h**). Graphs show means ± SEM, single neuron data points from four independent preparations; not significant, two-tailed Student’s *t*-test. **i** Representative images of primary SynI KO hippocampal neurons treated with either control CSF or LE- CSF (1.5 μg/mL) for 72 h and stained for β-III tubulin (blue), VGLUT1 (green), and VGAT (red). Scale bars, 10 and 1 μm for low and high magnification, respectively. **j** Density of VGLUT1- positive excitatory synapses (upper panel) and VGAT-positive inhibitory synapses (lower panel) in control and patient CSF-treated SynI KO neurons. Cumulative plots show pooled data from all neurons; graphs show means ± SEM, single neuron data points from three independent preparations; not significant, two-tailed Student’s *t*-test.

Although SynI is an intracellular antigen that never gets exposed on the cell surface in healthy neurons, we asked whether the effects of CSF containing SynI autoantibodies could be mediated by the interaction of autoantibodies with endogenously expressed SynI protein. To test this hypothesis, we repeated the above experiments by exposing SynI KO neurons to patient CSF and evaluating the effect on excitatory and inhibitory synaptic transmission. Unlike wild type neurons, SynI KO hippocampal cultures did not show any difference in frequency, amplitude and kinetics of both mEPSCs (Fig. 3a–d) and mIPSCs (Fig. 3e–h) when treated with either LE or control CSF. Consistent with these findings, there was no difference in the density of both excitatory and inhibitory synapses in SynI KO hippocampal neurons treated with LE-CSF with respect to those treated with control CSF (Fig. 3i, j). Altogether, these results indicate that patient CSF-induced dysregulation in primary neurons strictly targets the endogenous SynI protein.

### Internalization of SynI antibodies is mediated by clathrin-dependent endocytosis via Fc II/III receptors

**Fig. 4:**
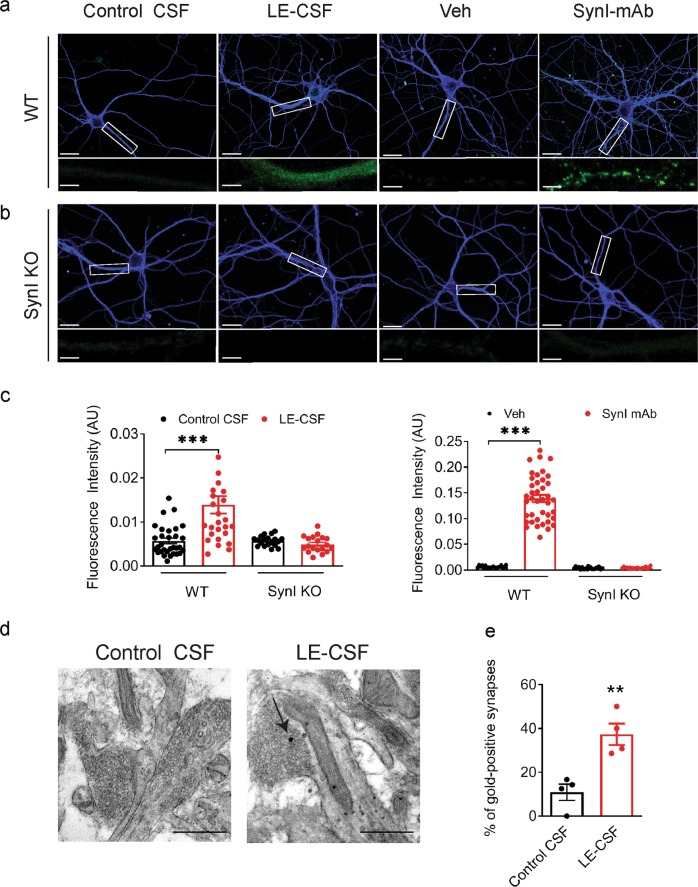
Patient autoantibodies and affinity-purified SynI antibodies have a similar distribution in primary neurons and their intracellular accumulation requires SynI. Representative images of WT (**a**) or SynI KO (**b**) neurons incubated for 72 h with either LE-CSF, SynI-mAb or equal volumes of the respective vehicles. Neurons were fixed and primary antibodies were visualized with anti-human and anti-mouse fluorescently conjugated secondary antibodies (green), respectively. Double staining for βIII tubulin (blue) was performed after fixation. Scale bars, 10 and 1 μm for low and high magnification, respectively. **c** Quantification of the intensity of primary antibody immunoreactivity. The graph shows means ± SEM, single neuron data points from 4 independent preparations; ***p < 0.001, two-way ANOVA/Bonferroni’s tests. **d** Representative TEM images of nerve terminals from cultured hippocampal neurons incubated with either control CSF or LE-CSF and processed for immunogold against human IgG antibody. Arrow indicates gold particles after gold enhancement. Scale bars, 200 nm. **e** Histograms representing the percentage of gold-positive synapses. Graphs show means ± SEM, *n* = 4–5 synapses from two independent preparations; *p < 0.05, two-tailed Student’s *t*-test.

To detail the mechanism responsible for SynI antibody-mediated effects, we asked whether anti-SynI antibodies enter neurons. To this aim, cells were treated with either LE-CSF or SynI-mAb for 72 h, fixed, permeabilized and immunoreacted with anti-human or anti-mouse secondary antibodies, respectively (Fig. 4a). Patient CSF-treated cells showed an intense immune staining of neuronal bodies and dendrites compared to control CSF, and an even stronger staining pattern was observed after incubation with SynI-mAb (Fig. 4a, c). In contrast, SynI KO neurons showed only a faint background signal under both conditions (Fig. 4b, c). Consistent with the strict intracellular location of SynI, no signal was detected without permeabilization (data not shown;^8^). The cell internalization of SynI autoantibodies was confirmed by ultrastructural analysis of nerve terminals subjected to pre-embedding immunogold electron microscopy. LE-CSF treated neurons showed a clear accumulation of gold particles directed against human IgG antibodies at synaptic terminals as compared with cultures treated with control CSF (Fig. 4d, e).

**Fig. 5:**
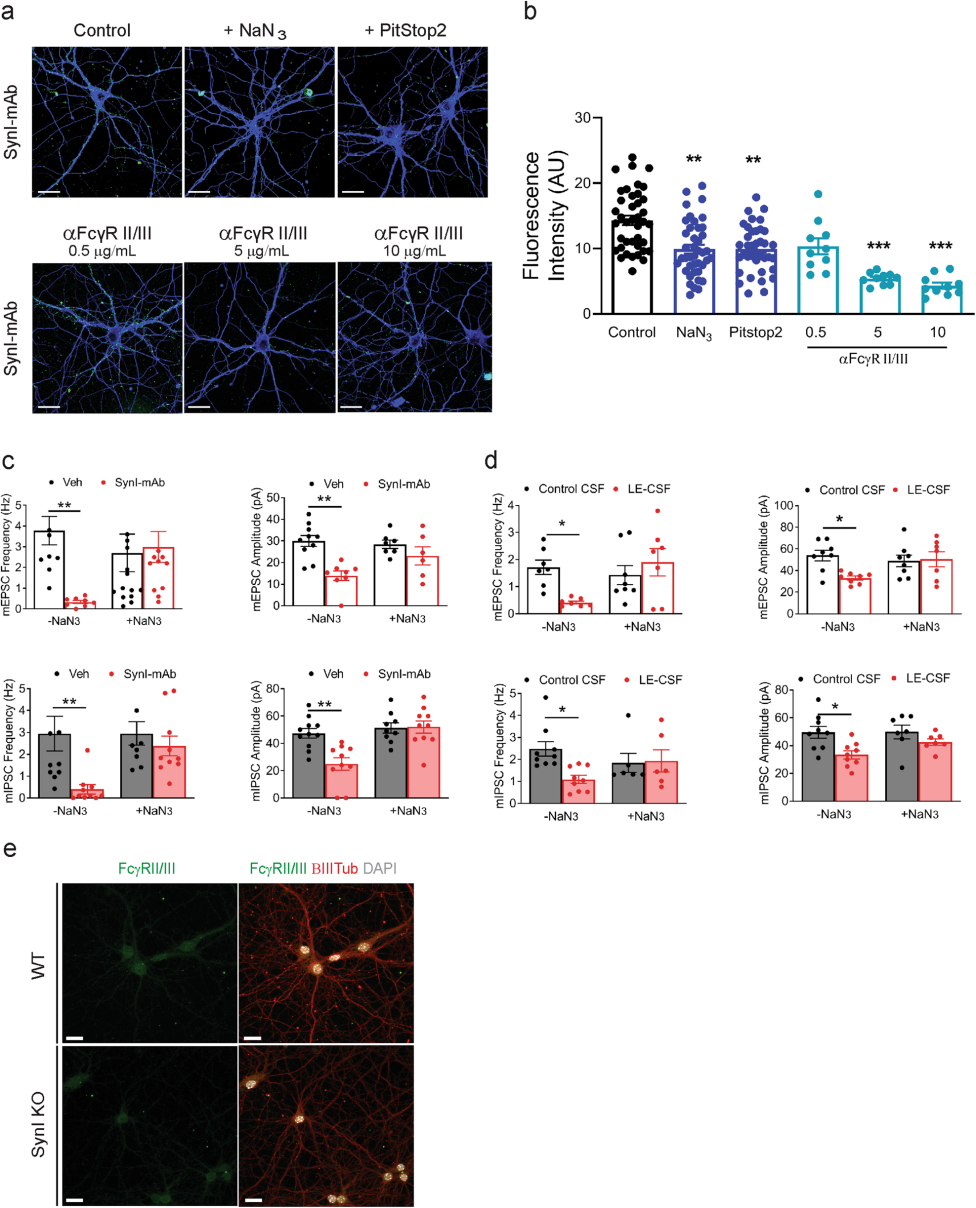
Blockade of clathrin-mediated endocytic pathway via Fc II/III receptors inhibits SynI antibody uptake and restores synaptic transmission. **a** Representative images of SynI-mAb (1.5 μg/mL)-treated hippocampal neurons incubated either with sodium azide (NaN_3_; 500 nM), PitStop 2 (30 μM) or α FcγR II/III antibody at the indicated concentrations for 72 h. The control condition indicates cells exposed to SynI-mAb in the presence of the NaN_3_/PitStop2 solvent. After treatments, cells were fixed, permeabilized and incubated with anti-mouse fluorescent secondary antibodies (green). Double staining for βIII-tubulin (blue) was performed after fixation. Scale bars, 10 μm. **b** Quantification of the immunoreactivity intensity of internalized SynI-mAb. Graph shows mean ± SEM, single neuron data points independent preparations; **p < 0.01, ***p < 0.001, one-way ANOVA/Dunnett’s tests versus control. **c** Mean frequency and amplitude of mEPSCs (upper panels) and mIPSCs (lower panels) in hippocampal neurons treated with either SynI-mAb (1.5 μg/mL) or vehicle for 72 h in the presence or absence of 500 nM NaN_3_. **d** Mean frequency and amplitude of mEPSCs (upper panels) and mIPSCs (lower panels) in hippocampal neurons treated with either control CSF or patient #1 CSF (1.5 μg/mL) for 72 h in the presence or absence of 500 nM NaN_3_. Graphs show means ± SEM, single neuron data points from three independent preparations; *p <0.05, **p < 0.01, two-way ANOVA/Bonferroni’s tests. **e** Expression of FcγII/III receptors in WT and SynI KO hippocampal neurons. Representative images of primary WT and SynI KO hippocampal neurons at 14.

In contrast to autoantibodies directed to membrane-bound receptors, it has been long discussed whether and how antibodies against intracellular antigens could reach their target. Thus, we tested the hypothesis that the uptake of anti-SynI antibodies was mediated by the endocytic pathway. Addition of the nonspecific blocker of ATP-dependent endocytosis sodium azide^16^ to neurons incubated with SynI-mAb induced a significant reduction of the antibody uptake (Fig. 5a, b) and abolished the effects of both SynI-mAb and LE-CSF treatments on mEPSC and mIPSC frequency and amplitude (Fig. 5c, d). To confirm this result using a distinct inhibitor of endocytosis, we pretreated neurons PitStop2, an inhibitor of clathrin-mediated endocytosis^17^. We found that PitStop2, similarly to sodium azide, reduced the internalization of SynI-mAb (Fig. 5a, b), indicating that the antibody uptake mainly occurs via clathrin-dependent endocytosis.

Fcγ receptors (FcγRs) are a family of molecules expressed on the surface of a variety of cells, including neurons, which bind the Fc region of IgG and are similarly expressed in both WT and SynI KO neurons^18^ (Fig. 5e). A number of studies have shown that the interaction between autoantibodies and FcγRs has a detrimental role in neurodegenerative pathologies, including Parkinson’s and Alzheimer’s diseases^19^. In particular, it has been shown that FcγRs can mediate antibody uptake into neurons via clathrin-mediated endocytosis^20^. To further clarify the molecular mechanism responsible for antibody uptake, we co-incubated hippocampal neurons with SynI-mAb and increasing concentrations of FcγR II/III receptor blocker anti-CD16/CD32 monoclonal antibody (α FcγR II/III). The extent of internalization of SynI-mAb, slightly inhibited by the lowest dose of αFcγR II/III antibodies (0.5 µg/mL), was strongly inhibited at higher concentrations of αFcγR II/III antibodies (5 and 10 µg/mL) as compared to the control condition (absence of α FcγR II/III antibody) (Fig. 5b, c). Co-treatment with unrelated isotype-matched anti-His-tag antibody did not affect the internalization of SynI-mAb (data not shown).

Altogether, these results strongly suggest that SynI-mAb uptake into neurons occurs via clathrin-dependent FcγR-mediated endocytosis.

### Endocytosed SynI antibodies can escape the endocytic compartments and target endogenous SynI

To analyze whether the extracellularly applied anti-SynI antibodies that had been endocytosed are released in the cytosol, we exposed primary neurons to the SynI-mAb antibody for 72 h and performed a gentle subcellular fractionation under isotonic high ionic strength conditions known to dissociate SynI from SVs and maintain the integrity of intracellular organelles^21,22^.

**Fig. 6:**
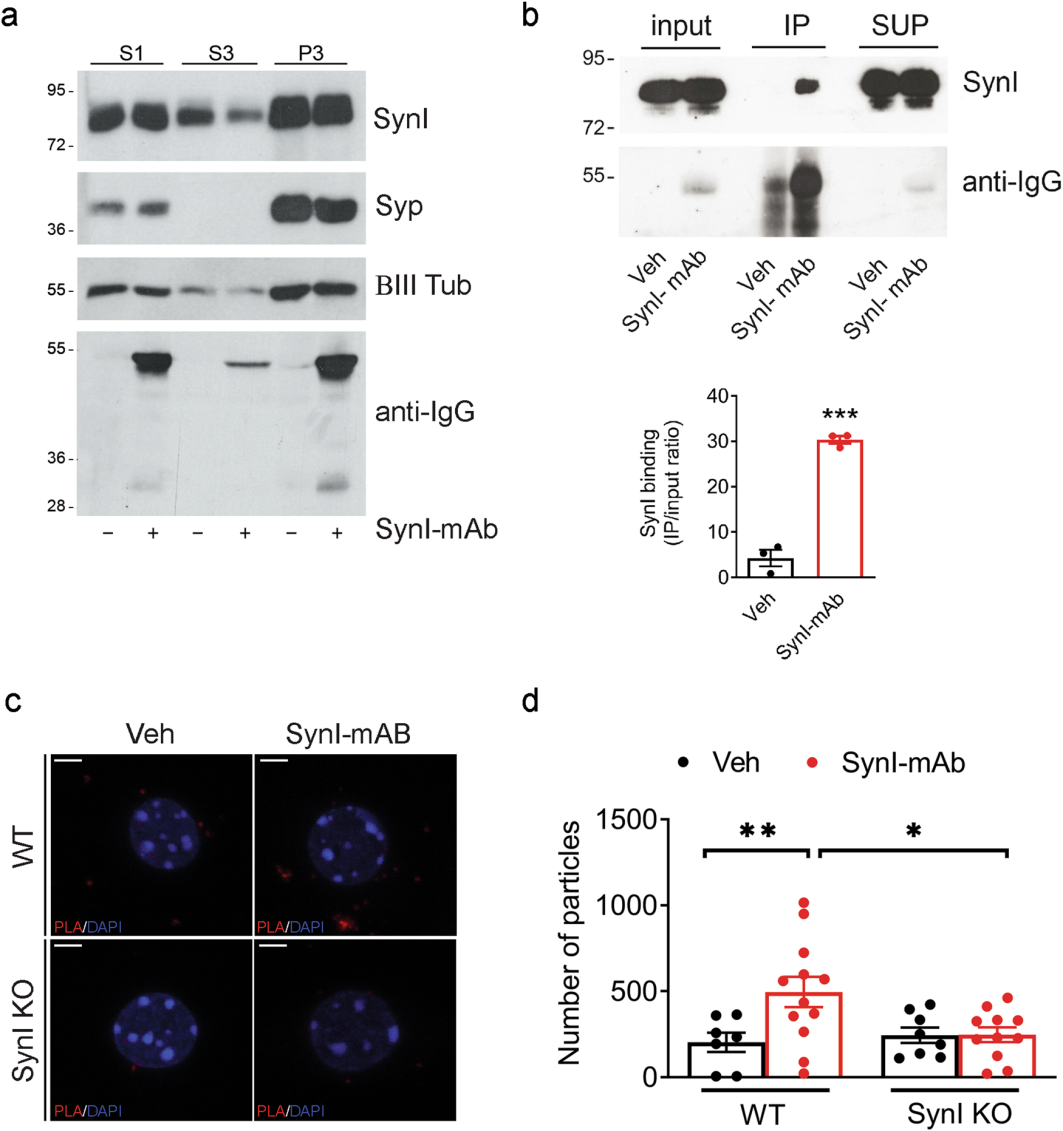
Endocytosed SynI antibodies escape the endocytic compartments and target endogenous SynI. **a** Hippocampal neurons, incubated daily for three days with either SynI-mAb (1.5 μg/mL) or vehicle, were subjected to subcellular fractionation to obtain the post-nuclear supernatant (S1), a cytosolic fraction (S3) and a membrane-enriched fraction (P3). A representative immunoblot with anti-SynI (SynI), anti-synaptophysin (Syp), anti-β-tubulin III (βIII Tub), and anti-mouse secondary antibodies (anti-IgG) to test the presence of the internalized SynI-mAb is shown. **b** Immunoprecipitation (IP) of the complex between internalized SynI antibodies and endogenous SynI was performed in hippocampal neuronal extracts with Protein G-Sepharose after incubation of neurons with either SynI-mAb or vehicle for 24 h. Equal aliquots of the starting material (input) and of the supernatants (SUP) together with the IP samples were subjected to western blotting with anti-SynI antibodies (SynI) and anti-mouse secondary antibodies (anti-IgG) to test the recovery of the internalized SynI antibodies. Left: A representative immunoblot is shown. Right: quantification of the SynI immunoreactivity signal in the immunoprecipitated samples, normalized to the binding in the control sample (means ± SEM, *n* = 3 independent experiments). Input, 20 μg cell extract. ***p < 0.001, two-tailed Student’s *t*-test. **c** Representative confocal images of in situ proximity ligation assay (PLA) in WT and SynI KO hippocampal neurons incubated with either SynI-mAb (1.5 μg/mL) or vehicle for 72 h. After fixation, cells were stained with Syn I G143 antibody and processed to detect SynI-mAb and synapsin I proximity interactions. Positive interactions are indicated as red dots. DAPI-stained nuclei are indicated in blue. Scale bars, 5 μm. **d** Quantification of the number of PLA spots. Graphs show means ± SEM, single neuron data points from three independent preparations; *p < 0.05, **p < 0.01, two-way ANOVA/Bonferroni’s tests.

Thus, we subjected the post-nuclear supernatant to high-speed centrifugation to separate membranes, organelles and cytoskeletal polymers (P3 fraction) from the soluble cytosolic fraction (S3). Interestingly, a detectable amount of the antibody was present in the S3 fraction, testifying that it escaped from the endocytic compartment (Fig. 6a).

To test whether endocytosed anti-SynI antibodies form an intracellular complex with endogenous SynI, we applied two experimental strategies. First, we performed an immunoprecipitation assay in wild type neurons extracellularly exposed to the SynI-mAb antibody for 72 h. When the SynI-mAb was quantitatively pulled down from the cell extract with Protein-G beads, a detectable amount of endogenous SynI was co-immunoprecipitated, indicating the formation of an intracellular complex between the exogenous antibody and SynI (Fig. 6b). Second, a physical interaction between the SynI-mAb and endogenous SynI was detected by the proximity ligation assay (PLA). WT and SynI KO neurons were treated with either vehicle or the SynI-mAb antibody for 72. After fixing, an affinity-purified rabbit antibody against the N-terminal domain of SynI, G143, was used to recognize endogenous SynI. Next, secondary antibodies coupled to different oligonucleotides (PLA rabbit and PLA mouse probes) were applied and the fluorescent PLA signal (red dots) generated by rolling-circle amplification was quantified. SynI-mAb-treated WT neurons showed abundant PLA dots compare to control conditions, whereas SynI KO neurons showed only few PLA puncta under both conditions (Fig. 6c, d). These data demonstrate that intracellular SynI antibodies physically interact with the endogenous target.

### Release of anti-SynI antibodies in the cytosol impairs SynI-dependent SV clustering

**Fig. 7:**
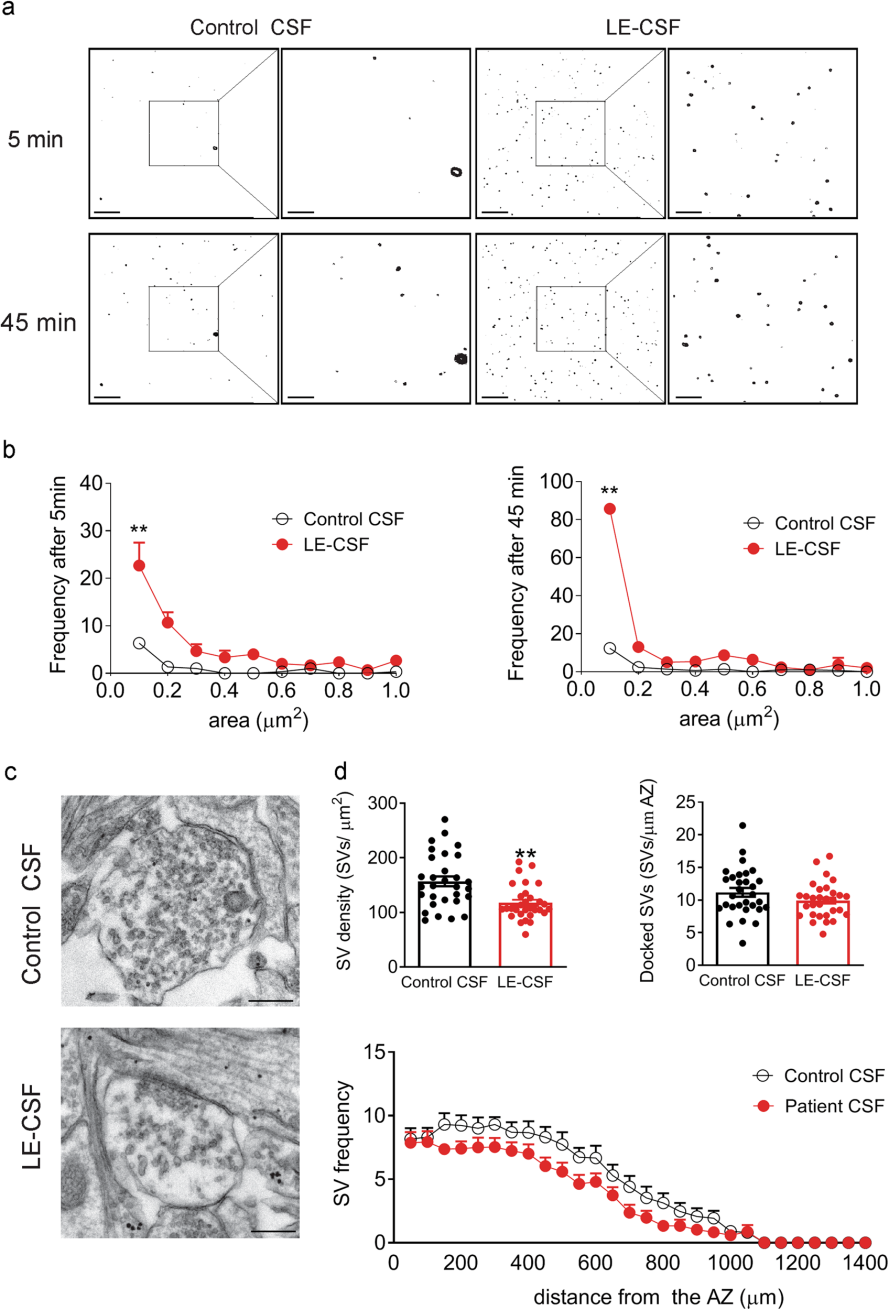
The interaction between internalized SynI antibodies and SynI decreases SV density and increases SynI liquid phase separation. **a**, **b** Representative images (**a**) and frequency distribution (**b**) of GFP-SynI droplets incubated with either control CSF or LE-CSF (25 μg/ml) for 5 and 45 min. Scale bars, 10 and 0.05 μm for low and high magnification, respectively. Each condition was assessed by two independent incubations. **c** Representative micrograph showing synapses from primary hippocampal neurons incubated with either control CSF or LE-CSF (1,5 μg/ml) for 72 h. Scale bar, 300 nm. **d** Upper panels: Quantification of SV and docked SV density in the analyzed terminals. Lower panel: quantification of the spatial distribution of SVs from active zone (AZ). Graphs show means ± SEM, single neuron data points from three independent preparations **p < 0.01, Mann–Whitney test.

It was recently demonstrated that SynI undergoes phase separation and condensate in droplets under physiological conditions in vitro, suggesting that SynI can guide protein phase separation required for SV clustering^23^. To assess whether exposure to SynI autoantibodies alters the ability of SynI to undergo phase separation and form droplets, we incubated purified GFP-SynI under conditions mimicking the intracellular milieu and analyzed the fluorescence signal by TIRF microscopy. After few minutes, GFP-SynI incubated with control CSF formed droplets and the process reached a plateau phase after 45 min, however clear and significant increase in droplet formation was observed after the exposure to patient’s CSF at both short and long time points (Fig. 7a, b).

If droplet formation reflects the state of aggregation of SynI and acts as sort of decoy mechanism for SynI during the SV cycle, one would expect a difference in the density of SVs in nerve terminals of neurons treated with LE-CSF, mimicking the effect of SynI deletion in mice^24^.

To ascertain this possibility, we performed an ultrastructural analysis of nerve terminals by conventional transmission electron microscopy in patient CSF-treated neurons. Interestingly, nerve terminals of patient CSF-treated neurons were depleted of SVs, with a significant reduction in density of SVs compared to control CSF-treated neurons, in the absence of changes in the AZ-docked SVs (Fig. 7c, d). This phenotype resembles the phenotype of SynI KO neurons^25–27^ and indicates that SynI autoantibodies sequester endogenous SynI and alter its ability to form liquid phase and cluster SVs.

## Discussion

Antibodies to cell adhesion molecules, neurotransmitter receptors, ion channels, proteins related to synaptogenesis, neurotransmitter synthesis and SV endocytosis have been identified in numerous group of neurological diseases^1^. Several studies have shown that antibodies to surface antigens have a clear pathogenic role in disease development and progression, whereas straightforward pathogenetic mechanisms are more difficult to identify for autoantibodies directed to intracellular antigens.

We have recently identified intrathecally synthesized IgA/IgG autoantibodies to the SV-associated protein SynI in several patients suffering from various neuropsychiatric diseases, including limbic encephalitis, multiple sclerosis, depression, anxiety, and bipolar disorders^13,14,23^. SynI is a SV phosphoprotein playing multiple roles in synaptic transmission and plasticity. It participates in synapse formation and maintenance, plays a major role in clustering SVs creating a RP of SVs and regulating their availability for exocytosis, and differentially affects the post-docking steps of release in excitatory and inhibitory synapses^8^. It is currently believed that its action in the assembly of the SV RP occurs through oligomerization of Syn molecules that cross-link SVs and bind them to the actin cytoskeleton^28–32^. Moreover, it was recently reported that SynI-induced phase separation in the cytoplasm of the nerve terminal restricts the formation of SV clusters in the proximity to presynaptic release sites^23,33^.

In this study, we provide evidence that human SynI autoantibodies can adversely contribute to synaptic dysfunction. Firstly, these antibodies alter excitatory and inhibitory synaptic transmission in cultured hippocampal neurons and decrease the levels of SynI-positive synaptic contacts. The effects are highly specific for SynI, as SynI KO neurons incubated with patient-derived CSF did not show any change in synaptic transmission. Moreover, an identical synaptic phenotype was observed using affinity-purified anti-SynI antibodies. The observed phenotype confirms the role of SynI in the formation and maintenance of synaptic connections, as well as in adjusting the number of synaptic connections in response to extracellular signals^34,35^

Secondly, these antibodies are internalized by a clathrin-dependent endocytosis mediated by FcγII/III receptors, which bind the constant region of IgG and are highly expressed in neurons. These findings are consistent with a general role of FcγRs in antibody uptake, as previously shown for anti-Tau antibody internalization in a mouse model of Alzheimer’s disease and for IgGs from amyotrophic lateral sclerosis patient in spinal cord motor neurons^20,36^. The neuronal uptake of the antibodies and the emergence neuronal phenotype are causally related, since inactivation of distinct mechanisms of endocytosis or neutralization of FcγRs largely abolished both the antibody uptake and the synaptopathy. Interestingly, no antibody accumulation was observed in SynI KO neurons. We speculate that antibody uptake is equally efficient in both genotypes, but that, once internalized into SynI KO neurons, SynI autoantibodies cannot bind the endogenous target and are therefore more prone to degradation. Once internalized, the exogenous antibodies escape the endocytic compartment and accumulate in the cytosol, where they bind endogenous SynI, as demonstrated by subcellular fractionation, immunoprecipitation and in situ PLA assays.

Recent studies have shown liquid–liquid phase separation is a mechanism through which intracellular components can assemble into membrane-lacking compartments^33,37–40^ and that SynI, present in high concentrations within nerve terminals, can undergo phase separation leading to SV clustering^23^. Indeed, we found that the formation of a SynI/antibody complex after incubation with patient CSF increases droplet formation, reflecting an excessive SynI aggregation/sequestration that may impair clustering of SynI and induce their dispersion in the cytosol. Notably, treatment of neurons with patient CSF induced a synaptic phenotype characterized by decreased overall SV density with no change in the number of docked SVs that fully mimics the phenotype of SynI KO mice^25–27^. These results are consistent with the hypothesis that anti-SynI antibodies determine a loss-of-function effect in primary neurons and also agree with the dramatic loss of SV clusters following injection of anti-SynI antibodies in giant reticulo-spinal axons of the lamprey^41^.

In the diseases associated with GAD65 and amphiphysin autoantibodies, several findings have shown the pathogenic role of the autoantibodies^3–5^. Since both intracellular antigens are involved in the SV exo-endocytotic cycle, one possibility is that they get transiently exposed to the extracellular environment during synaptic activity. This model could also account for SynI antibody uptake. Such a mechanism has been proposed for the nerve terminal uptake of Botulinum neurotoxins that bind to the intraluminal domains of the integral SV proteins SV2 and synaptotagmin that become exposed during exocytosis^42^. However SynI, similarly to GAD and amphiphysin, is not an integral membrane protein and coats the cytosolic side of the SV membrane, so that it should never get exposed to the extracellular space during the SV cycle. Although the highly hydrophobic C domain of SynI partially penetrates the hydrophobic core of the SV membrane, it is highly unlikely that it can cross the whole membrane thickness^22,43^. In addition, the SynI autoantibodies found in limbic encephalitis and clinically isolated syndrome patients are directed to the hydrophilic and highly immunogenic COOH-terminal domain that has a permanent cytosolic exposure^15^. It is noteworthy to mention that the SynI COOH-terminal D-domain, target of epileptogenic mutations in humans^9,44^, plays a key role in SynI function: it participates in dimerization and hosts two CaM kinase II sites, whose phosphorylation regulates SynI binding to actin and SVs, as well as its phase separation properties^15,23,31,45^.

The etiology and pathogenesis of autoantibody-mediated brain diseases is only partially understood, as far as the antibody production trigger in the non-paraneoplastic cases and the antibody effects are concerned. It is possible that the intrathecal production of SynI autoantibodies is triggered by neuronal damage or by extracellular release of SV-bound SynI via exosomes under conditions of high neuronal activity or oxidative stress^46^. While their presence may be an epiphenomenon of cell damage induced by the underlying neuronal pathology, the secondary production of SynI autoantibodies can play a role in disease progression and pleiotropy clinical symptoms (see refs. ^47,48^ for review). Our work has the limitation of a small number of human samples and this frequently occurs in the earliest description of new clinical findings. However, our report provides a first proof of principle for the detrimental effects of anti-SynI antibodies in autoimmune diseases and may provide new insights in other forms of neurological pathologies associated with autoantibodies to intracellular self-antigens.

## Materials and methods

### Human CSF samples

CSF samples were obtained by lumbar punctures, which were performed for diagnostic purposes only and with the patients’ written informed consent. Following routine diagnostic work-up, remaining CSF was stored at −20 °C. The study was approved by the Institutional Review Board of Charité - Universitätsmedizin Berlin (EA1/083/15 and EA1/182/10) and participants provided written informed consent. Demographics and clinical features of the patients included in this work are summarized in Supplementary Table S1.

### Preparation of primary neurons

Primary hippocampal cultures were prepared from wild type C57Bl/6J mice (Charles River, Calco, Italy) and SynI KO mice extensively backcrossed in the C57Bl/6J background, as previously described^10^. All experiments were carried out in accordance with the guidelines established by the European Community Council (Directive 2010/63/EU of 22 September 2010) and were approved by the Italian Ministry of Health. For experiments involving treatments with human CSF (indicated as LE-CSF for limbic encephalitis patient, SM-CSF for multiple sclerosis patient and control CSF for healthy patients) and affinity-purified SynI antibody (SynI-mAb, monoclonal mouse anti-Synapsin I antibody directed against the SynI 435–475 peptide in the D domain of SynI; #106011, Synaptic System, Germany), cultures were treated daily for 3 days (11–14 DIV) with a medium containing either 1.5 μg IgG/mL CSF or SynI-mAb antibody. As a control for the treatment with SynI-mAb, cultures were subjected to the same medium change with addition of an equivalent volume of vehicle. For the experiments aimed at determining the role of endocytosis on SynI antibody internalization, 500 nM sodium azide (NaN_3_; Sigma-Aldrich, Milano, Italy), 30 µM Pitstop2 (Abcam, Cambridge, MA), CD16/CD32 rabbit monoclonal antibody (clone 2.4G2, ABS410, Enzo LifeScience, New York, NY) or 6X His-tag rat monoclonal antibody (ab206504, Abcam) was added 30 min before the treatments.

### Patch-clamp electrophysiology

Primary mouse hippocampal neurons incubated under the various conditions were used for patch-clamp recordings at 14 DIV as previously described^49^. Miniature PSCs (mPSCs) were acquired at 10 to 20 kHz sample frequency, filtered at half the acquisition rate with an 8-pole low-pass Bessel filter, and analyzed by using the Minianalysis program (Synaptosoft, Leonia, NJ). Amplitude and frequency of mPSCs were calculated using a peak detector function using different appropriate threshold amplitude and area. All parameters were analyzed using pClamp (Molecular Devices) and Prism7 (GraphPad Software, Inc.) software.

### Immunofluorescence

Hippocampal neurons were stained as described^50^. The primary antibodies used were anti-Synapsin I (clone 10.22, Millipore, USA), anti-vesicular glutamate transporter-1 (VGLUT1, AB5905, Millipore), anti-vesicular GABA transporter (VGAT, 131 011, Synaptic System), anti-β-tubulin III (T2200, Sigma-Aldrich), anti-green fluorescent protein (GFP, 11814460001, Sigma-Aldrich), anti-FcγRII/III (clone 2.4G2, ABS410, Enzo LifeScience). Incubation with primary antibodies (patient/control CSF and SynI-mAb) to visualize anti-SynI antibody internalization was performed in live neurons. After the primary incubation, neurons were incubated for 45 min with secondary antibodies in blocking buffer. Fluorescently conjugated secondary antibodies were from Molecular Probes (Thermo-Fischer Scientific). Image acquisitions were performed using a confocal microscope (SP8, Leica Microsystems, Wetzlar, Germany) at ×63 (1.4 NA) magnification. Z-stacks were acquired every 300 nm, 10 fields/sample. SynI, VGLUT and VGAT fluorescence intensity values were normalized to the relative cell volume calculated on the basis of βIII-tubulin labeling. The analysis was conducted using ImageJ software (ver. 1.51 k). For each set of experiments, all images were acquired using identical exposure settings.

### Transmission electron microscopy

Primary hippocampal neurons were processed as described previously^10^. For each experimental condition, at least 30 images of synapses were acquired at 10,000x magnification (sampled area per experimental condition: 36 μm^2^). Synaptic morphological features, including nerve terminal area, active zone (AZ) length, number and density of total and AZ-docked SVs, distance from the AZ, number and density of gold particles were determined using ImageJ software (ver. 1.51 k). SVs were defined as vesicles with a diameter of 40–50 nm.

### Subcellular fractionation and immunoprecipitation assays

Hippocampal neurons were treated daily for 3 days (11–14 DIV) with 1.5 µg/mL SynI-mAb in cell medium and subjected to biochemical fractionation. Cells were homogenized in PBS/200 mM NaCl supplemented with 1 mM phenylmethylsulfonyl fluoride (PMSF)/1 mM pepstatin (Sigma-Aldrich), incubated for 1 h on ice and cleared by low-speed centrifugation (10 min at 10,000 × *g* at 4 °C). The post-nuclear supernatant (S1) was centrifuged at 95,000 rpm for 1 h (Beckman TLA 100.2 rotor) to obtain a cytosolic fraction (S3) and a membrane-enriched fraction (P3). For immunoprecipitation assays, neurons, incubated with 1.5 µg/mL SynI-mAb for 72 h in cell medium, were lysed in lysis buffer (150 mM NaCl, 50 mM Tris-HCl pH 7.4, 1 mM EDTA, 1% Triton X-100) supplemented with 1 mM PMSF/1 mM pepstatin. After 10 min incubation on ice, lysates were collected and clarified by centrifugation (10 min at 10,000 × *g* at 4 °C). Equivalent amounts of cell extract (500 µg) were incubated for 2 h at 4 °C with Protein G-Sepharose (GE Healthcare) and the samples were then extensively washed in lysis buffer. Protein concentration of the samples was determined by the Bradford Assay (Bio-Rad) and equivalent amounts of protein were subjected to SDS-PAGE and western blotting with the following primary antibodies: rabbit anti-SynI (5297, Cell Signaling), rabbit anti-synaptophysin (10101, Synaptic System), rabbit anti-β-tubulin III (T2200, Sigma-Aldrich) followed by peroxidase-conjugated goat anti-rabbit secondary antibodies (Bio-Rad, USA). The presence and efficient immunoprecipitation of SynI-mAb was directly revealed by incubation of the nitrocellulose membrane with peroxidase-conjugated goat anti-mouse secondary antibodies (Bio-Rad). Bands were revealed with the ECL chemiluminescence detection system (Thermo Scientific) and quantified by densitometric analysis of the fluorograms.

### Indirect proximity ligation assay (PLA)

The in situ PLA was performed on WT and SynI KO neurons treated daily for 3 days (11–14 DIV) with a medium containing either 1.5 μg SynI-mAb antibody or vehicle. Cells were fixed PBS-4% paraformaldehyde for 15 min at room temperature (RT), permeabilized with 0.1% Triton X-100 for 5 min. DuoLink PLA technology probes and reagents (DUO92008, Sigma-Aldrich) were used as described^51^. Two affinity-purified rabbit antibodies against SynI were used to recognize endogenous SynI: SynI G143 (directed against the SynI 3–13 peptide in the A domain^52^; SynI G115 (directed against the SynI 587–609 peptide in the D domain of SynI^45^. Coverslips were mounted with Duolink mounting media with DAPI. Proximity ligation assay imaging was performed within 6 h using a confocal microscope (SP8, Leica Microsystems) at 63× (1.4 NA) magnification. The number of puncta per image was calculated using ImageJ (ver. 1.51 k). For each set of experiments, all images were acquired using identical exposure settings.

### GFP-SynI protein expression and purification

The expression vector for GFP-tagged rat SynI (a-isoform; GFP-SynI) was kindly donated by H.-T. Kao (Brown University, Providence, RI). GFP-Syn I was expressed in HEK293T cells using calcium phosphate (40 μg GFP-SynI for 3.5 × 10^6^ cells/150 mm dish). HEK293T cells were routinely cultured in IMDM (Sigma-Aldrich), supplemented with 100 units/ml penicillin, 100 μg/ml streptomycin, glutamine, and 10% heat-inactivated FCS (Life Technologies). HEK293T cells were lysed in buffer that contained 25 mM Tris-HCl (pH 7.4), 300 mM NaCl, 0.5 mM Tris-2-carboxyethyl-phosphine hydrochloride (TCEP), and protease inhibitors (1 mM PMSF/1 mM pepstatin; Sigma-Aldrich). The lysate was centrifuged for 1 h at 17,000 g, followed by affinity purification of GFP-SynI using GFP-Trap A (Chromotek, Germany) for 2 h. After extensive washes in washing buffer (25 mM Tris-HCl (pH 7.4), 150 mM NaCl, 0.5 mM TCEP), bound proteins were eluted by 250 μL of 0.2 M glycine pH 2.5 followed by 30 s incubation under constant mixing and centrifugation. The supernatant was immediately neutralized by adding 25 μL of Tris-base (pH 10.4) and stored at −80 °C until use.

### Total internal reflection fluorescence microscopy

Droplets of GFP-SynI (1 μM) were prepared in 25 mM Tris-HCl (pH 7.4), 150 mM NaCl, 0.5 mM TCEP, 3% PEG 8000 (Fluka Chemicals) in a final volume of 100 μl. Control CSF, patient CSF or water, in volumes equivalent to 3 μg patient’s IgG, was added to a final volume of 120 μL. For total internal reflection fluorescence microscopy (TIRFM), the final mixture was immediately pipetted on 35 mm-glass bottom dishes (P35G-0-14-C, MatTek Corp, USA) pre-coated with poly-L-lysine (1 mg/ml, Sigma). TIRFM images were acquired at RT using AF6000LX/TIRF MC at ×100 magnification (PL FLUOTAR HCX N.A. 1.30-0.60 OIL 1.4 NA) (Leica) and processed using Image J software (ver. 1.51 k).

### Data analysis

Results are presented as means ± SEM. All experiments were independently performed at least twice. No statistical methods were used to determine the sample size for experiments. Outliers were determined via Grubb’s test. Normal distribution of data were assessed using the Kolmogorov−Smirnov test. The two-tailed unpaired Student’s *t*-test was used to compare two normally distributed sample groups, while either one- or two-way ANOVA followed by Bonferroni’s multiple comparison test was used to compare more than two normally distributed sample groups. For datasets of non-normal distribution, Mann–Whitney test or Kruskal–Wallis and Dunn’s post hoc comparison were used. A *p* < 0.05 was considered significant. Statistical analysis was carried out using SigmaStat 13 (Systat Software).

## Supplementary information

Supplemental Table 1

## References

[1] Williams JP, Carlson NG, Greenlee JE (2018). Antibodies in autoimmune human neurological disease: pathogenesis and immunopathology. Semin. Neurol..

[2] Dalmau J, Geis C, Graus F (2017). Autoantibodies to synaptic receptors and neuronal cell surface proteins in autoimmune diseases of the central nervous system. Physiol. Rev..

[3] Werner C (2016). Human autoantibodies to amphiphysin induce defective presynaptic vesicle dynamics and composition. Brain.

[4] Manto M (2015). Disease-specific monoclonal antibodies targeting glutamate decarboxylase impair GABAergic neurotransmission and affect motor learning and behavioral functions. Front. Behav. Neurosci..

[5] Solimena M (1988). Autoantibodies to glutamic acid decarboxylase in a patient with stiff-man syndrome, epilepsy, and type I diabetes mellitus. N. Engl. J. Med.

[6] Geis C (2010). Stiff person syndrome-associated autoantibodies to amphiphysin mediate reduced GABAergic inhibition. Brain.

[7] Sommer C (2005). Paraneoplastic stiff-person syndrome: passive transfer to rats by means of IgG antibodies to amphiphysin. Lancet.

[8] Cesca F, Baldelli P, Valtorta F, Benfenati F (2010). The synapsins: key actors of synapse function and plasticity. Prog. Neurobiol..

[9] Fassio A (2011). SYN1 loss-of-function mutations in autism and partial epilepsy cause impaired synaptic function. Hum. Mol. Genet.

[10] Lignani G (2013). Epileptogenic Q555X SYN1 mutant triggers imbalances in release dynamics and short-term plasticity. Hum. Mol. Genet.

[11] Guarnieri FC (2017). A novel SYN1 missense mutation in non-syndromic X-linked intellectual disability affects synaptic vesicle life cycle, clustering and mobility. Hum. Mol. Genet.

[12] Peron A, Baratang NV, Canevini MP, Campeau PM, Vignoli A (2018). Hot water epilepsy and SYN1 variants. Epilepsia.

[13] Piepgras J (2015). Intrathecal immunoglobulin A and G antibodies to synapsin in a patient with limbic encephalitis. Neurol. Neuroimmunol. Neuroinflamm..

[14] Holtje M (2017). Synapsin-antibodies in psychiatric and neurological disorders: prevalence and clinical findings. Brain Behav. Immun..

[15] Mertens R (2018). Epitope specificity of anti-synapsin autoantibodies: differential targeting of synapsin I domains. PLoS ONE.

[16] Schmid SL, Carter LL (1990). ATP is required for receptor-mediated endocytosis in intact cells. J. Cell Biol..

[17] von Kleist L (2011). Role of the clathrin terminal domain in regulating coated pit dynamics revealed by small molecule inhibition. Cell.

[18] Okun E, Mattson MP, Arumugam TV (2010). Involvement of Fc receptors in disorders of the central nervous system. Neuromolecular Med.

[19] Fuller JP, Stavenhagen JB, Teeling JL (2014). New roles for Fc receptors in neurodegeneration-the impact on immunotherapy for Alzheimer’s disease. Front. Neurosci..

[20] Congdon EE, Gu J, Sait HB, Sigurdsson EM (2013). Antibody uptake into neurons occurs primarily via clathrin-dependent Fcgamma receptor endocytosis and is a prerequisite for acute tau protein clearance. J. Biol. Chem..

[21] Huttner WB (1983). A nerve terminal-specific phosphoprotein. III. Its association with synaptic vesicles studied in a highly purified synaptic vesicle preparation. J. Cell Biol..

[22] Benfenati F, Bahler M, Jahn R, Greengard P (1989). Interactions of synapsin I with small synaptic vesicles: distinct sites in synapsin I bind to vesicle phospholipids and vesicle proteins. J. Cell Biol..

[23] Milovanovic D, Wu Y, Bian X, De Camilli P (2018). A liquid phase of synapsin and lipid vesicles. Science.

[24] Gitler D (2004). Different presynaptic roles of synapsins at excitatory and inhibitory synapses. J. Neurosci..

[25] Li L (1995). Impairment of synaptic vesicle clustering and of synaptic transmission, and increased seizure propensity, in synapsin I-deficient mice. Proc. Natl Acad. Sci. USA.

[26] Rosahl TW (1995). Essential functions of synapsins I and II in synaptic vesicle regulation. Nature.

[27] Takei Y (1995). Synapsin I deficiency results in the structural change in the presynaptic terminals in the murine nervous system. J. Cell Biol..

[28] Benfenati F, Valtorta F (1995). Neuroexocytosis. Curr. Top. Microbiol. Immunol..

[29] Esser L (1998). Synapsin I is structurally similar to ATP-utilizing enzymes. EMBO J..

[30] Hosaka M, Sudhof TC (1998). Synapsins I and II are ATP-binding proteins with differential Ca2+ regulation. J. Biol. Chem..

[31] Monaldi I (2010). The highly conserved synapsin domain E mediates synapsin dimerization and phospholipid vesicle clustering. Biochem. J..

[32] Orlando M (2014). Functional role of ATP binding to synapsin I in synaptic vesicle trafficking and release dynamics. J. Neurosci..

[33] Milovanovic D, De Camilli P (2017). Synaptic vesicle clusters at synapses: a distinct liquid phase?. Neuron.

[34] Perlini LE (2011). Effects of phosphorylation and neuronal activity on the control of synapse formation by synapsin I. J. Cell Sci..

[35] Fornasiero EF, Bonanomi D, Benfenati F, Valtorta F (2010). The role of synapsins in neuronal development. Cell Mol. Life Sci..

[36] Mohamed HA (2002). Immunoglobulin Fc gamma receptor promotes immunoglobulin uptake, immunoglobulin-mediated calcium increase, and neurotransmitter release in motor neurons. J. Neurosci. Res.

[37] Brangwynne CP (2013). Phase transitions and size scaling of membrane-less organelles. J. Cell Biol..

[38] Case LB, Zhang X, Ditlev JA, Rosen MK (2019). Stoichiometry controls activity of phase-separated clusters of actin signaling proteins. Science.

[39] Huang WYC (2019). A molecular assembly phase transition and kinetic proofreading modulate Ras activation by SOS. Science.

[40] Hyman AA, Weber CA, Julicher F (2014). Liquid-liquid phase separation in biology. Annu. Rev. Cell Dev. Biol..

[41] Pieribone VA (1995). Distinct pools of synaptic vesicles in neurotransmitter release. Nature.

[42] Ahnert-Hilger G, Munster-Wandowski A, Holtje M (2013). Synaptic vesicle proteins: targets and routes for botulinum neurotoxins. Curr. Top. Microbiol Immunol..

[43] Stefani G (1997). Kinetic analysis of the phosphorylation-dependent interactions of synapsin I with rat brain synaptic vesicles. J. Physiol..

[44] Nguyen DK (2015). X-linked focal epilepsy with reflex bathing seizures: Characterization of a distinct epileptic syndrome. Epilepsia.

[45] Benfenati F (1992). Synaptic vesicle-associated Ca2+/calmodulin-dependent protein kinase II is a binding protein for synapsin I. Nature.

[46] Wang S (2011). Synapsin I is an oligomannose-carrying glycoprotein, acts as an oligomannose-binding lectin, and promotes neurite outgrowth and neuronal survival when released via glia-derived exosomes. J. Neurosci..

[47] Crisp SJ, Kullmann DM, Vincent A (2016). Autoimmune synaptopathies. Nat. Rev. Neurosci..

[48] Fukata M, Yokoi N, Fukata Y (2018). Neurobiology of autoimmune encephalitis. Curr. Opin. Neurobiol..

[49] Bramini M (2016). Graphene oxide nanosheets disrupt lipid composition, Ca(2+) homeostasis, and synaptic transmission in primary cortical neurons. ACS Nano.

[50] Rocchi Anna, Moretti Daniela, Lignani Gabriele, Colombo Elisabetta, Scholz-Starke Joachim, Baldelli Pietro, Tkatch Tatiana, Benfenati Fabio (2019). Neurite-Enriched MicroRNA-218 Stimulates Translation of the GluA2 Subunit and Increases Excitatory Synaptic Strength. Molecular Neurobiology.

[51] Gomes I, Sierra S, Devi LA (2016). Detection of receptor heteromerization using in situ proximity ligation assay. Curr. Protoc. Pharm..

[52] Vaccaro P (1997). Anti-synapsin monoclonal antibodies: epitope mapping and inhibitory effects on phosphorylation and Grb2 binding. Brain Res. Mol. Brain Res..

