# Correction: Autoantibodies to synapsin I sequestrate synapsin I and alter synaptic function

**DOI:** 10.1038/s41419-019-2220-y

**Published:** 2020-01-14

**Authors:** Anna Rocchi, Silvio Sacchetti, Antonio De Fusco, Silvia Giovedi, Barbara Parisi, Fabrizia Cesca, Markus Höltje, Klemens Ruprecht, Gudrun Ahnert-Hilger, Fabio Benfenati

**Affiliations:** 1 Center for Synaptic Neuroscience and Technology, Istituto Italiano di Tecnologia, Largo Rosanna Benzi 10, 16132 Genova, Italy; 2 IRCSS, Ospedale Policlinico San Martino, Largo Rosanna Benzi 10, 16132 Genova, Italy; 3 Department of Experimental Medicine, University of Genova, Viale Benedetto XV, 3, 16132 Genova, Italy; 4 Department of Life Science, University of Trieste, via Giorgieri, 5, 34127 Trieste, Italy; 5 Institute of Integrative Neuroanatomy, Charité – Universitätsmedizin Berlin, Corporate member of Freie Universität Berlin, Humboldt-Universität zu Berlin, and Berlin Institute of Health, Berlin, Germany; 6 Department of Neurology, Charité – Universitätsmedizin Berlin, Corporate member of Freie Universität Berlin, Humboldt-Universität zu Berlin, and Berlin Institute of Health, Berlin, Germany

**Correction to: Cell Death and Disease**

10.1038/s41419-019-2106-zpublished online 14 November 2019

Since online publication of this article, the authors noticed that in the legend of Fig. 5, the legend to “panel e” was mistakenly omitted. The full text should read as follows:

**Fig. 5 a** Representative images of SynI-mAb (1.5 μg/mL)-treated hippocampal neurons incubated either with sodium azide (NaN_3_; 500 nM), PitStop 2 (30 μM) or α FcγR II/III antibody at the indicated concentrations for 72 h. The control condition indicates cells exposed to SynI-mAb in the presence of the NaN_3_/PitStop2 solvent. After treatments, cells were fixed, permeabilized and incubated with anti-mouse fluorescent secondary antibodies (green). Double staining for βIII-tubulin (blue) was performed after fixation. Scale bars, 10 μm. **b** Quantification of the immunoreactivity intensity of internalized SynI-mAb. Graph shows mean ± SEM, single neuron data points independent preparations; **p < 0.01, ***p < 0.001, one-way ANOVA/Dunnett’s tests versus control. **c** Mean frequency and amplitude of mEPSCs (upper panels) and mIPSCs (lower panels) in hippocampal neurons treated with either SynI-mAb (1.5 μg/mL) or vehicle for 72 h in the presence or absence of 500 nM NaN_3_. **d** Mean frequency and amplitude of mEPSCs (upper panels) and mIPSCs (lower panels) in hippocampal neurons treated with either control CSF or patient #1 CSF (1.5 μg/mL) for 72 h in the presence or absence of 500 nM NaN_3_. Graphs show means ± SEM, single neuron data points from three independent preparations; *p < 0.05, **p < 0.01, two-way ANOVA/Bonferroni’s tests. **e** Expression of FcγII/III receptors in WT and SynI KO hippocampal neurons. Representative images of primary WT and SynI KO hippocampal neurons at 14.

This has been corrected in the PDF and HTML versions of the article.

The authors apologise for any inconvenience caused.

