# Supplementary material for: Autoantibodies to synapsin I sequestrate synapsin I and alter synaptic function
Source: Cell Death Dis. 2019 Nov 14;10(11):864. doi: 10.1038/s41419-019-2106-z (PMC6856194; doi:10.1038/s41419-019-2106-z)
Supplement: Supplementary file 1 — Supplemental Table 1 [file 41419_2019_2106_MOESM1_ESM.doc]

**Supplementary Table 1**

**Demographic and clinical data of patients investigated in this study**

| **Patient ID** | **Age at lumbar puncture, years** | **Sex** | **clinical diagnosis** | **SynIa IgG titer serum**^a^ | **SynIa IgG titer CSF**^a^ | **SynIa IgA titer serum** | **SynIa IgA titer CSF** |
| --- | --- | --- | --- | --- | --- | --- | --- |
| LE-Patient^b^ | 69 | male | Limbic encephalitis | 1:3,200 | 1:3,200 | 1:1,000 | 1:1,000 |
| CIS-Patient^c^ | 24 | female | Clinically isolated syndrome | 1:10,000 | 1:1,000 | not detectable | not detectable |
| Control patient #1 | 63 | male | Normal pressure hydrocephalus | not detectable | not detectable | not detectable | not detectable |
| Control patient #2 | 19 | female | unspecific headache | not detectable | not detectable | not detectable | not detectable |

^a^Antibodies to SynIa were determined in patients’ serum and CSF by cell-based assays using HEK293 cells transfected with rat SynIa cDNA (Ref. Höltje et al. 2017 and Mertens et al. 2018).

^b^LE-Patient is the index patient in whom SynI Ab were originally identified. The clinical and paraclinical findings of this patient were previously reported in Piepgras et al. 2015.

^c^CSI-Patient is the same patient as patient CIS_0117, previously reported in Höltje et al. 2017 and Mertens et al. 2018.
